# Shapeshifting in the Venus flytrap (*Dionaea muscipula*): Morphological and biomechanical adaptations and the potential costs of a failed hunting cycle

**DOI:** 10.3389/fpls.2022.970320

**Published:** 2022-09-02

**Authors:** Grażyna M. Durak, Thomas Speck, Simon Poppinga

**Affiliations:** ^1^Plant Biomechanics Group, Botanical Garden, Department of Biology, University of Freiburg, Freiburg, Germany; ^2^Cluster of Excellence livMatS @ FIT, Freiburg Center for Interactive Materials and Bioinspired Technologies, University of Freiburg, Freiburg, Germany; ^3^Botanical Garden, Department of Biology, Technical University of Darmstadt, Darmstadt, Germany

**Keywords:** biomechanics, carnivorous plants, snap-traps, plant movement, functional morphology, hunting cycle

## Abstract

The evolutionary roots of carnivory in the Venus flytrap (*Dionaea muscipula*) stem from a defense response to plant injury caused by, e.g., herbivores. *Dionaea muscipula* aka. Darwin’s most wonderful plant underwent extensive modification of leaves into snap-traps specialized for prey capture. Even the tiny seedlings of the Venus flytrap already produce fully functional, millimeter-sized traps. The trap size increases as the plant matures, enabling capture of larger prey. The movement of snap-traps is very fast (~100–300 ms) and is actuated by a combination of changes in the hydrostatic pressure of the leaf tissue with the release of prestress (embedded energy), triggering a snap-through of the trap lobes. This instability phenomenon is facilitated by the double curvature of the trap lobes. In contrast, trap reopening is a slower process dependent on trap size and morphology, heavily reliant on turgor and/or cell growth. Once a prey item is caught, the trap reconfigures its shape, seals itself off and forms a digestive cavity allowing the plant to release an enzymatic cocktail to draw nutrition from its captive. Interestingly, a failed attempt to capture prey can come at a heavy cost: the trap can break during reopening, thus losing its functionality. In this mini-review, we provide a detailed account of morphological adaptations and biomechanical processes involved in the trap movement during *D. muscipula* hunting cycle, and discuss possible reasons for and consequences of trap breakage. We also provide a brief introduction to the biological aspects underlying plant motion and their evolutionary background.

## Introduction

The Venus flytrap (*Dionaea muscipula,* Droseraceae) is a subtropical, carnivorous plant (Schnell, 2002; Bailey and McPherson, 2012) widely known for producing snap-traps looking very much like sets of “green jaws.” Interestingly, the carnivorous habit of this plant builds on the defense systems against herbivory, facilitating the plant’s survival in the nutrient poor wetlands where they grow (Bailey and McPherson, 2012; Pavlovič et al., 2017; Fleischmann et al., 2018). The incredibly fast thigmonastic motion performed by the snap-traps of *D. muscipula* can be triggered by touching mechanosensitive hairs, making it a perfect trapping system for, e.g., small arthropods, which can then be digested and absorbed by the plant. To enable prey capture, *D. muscipula* underwent extreme leaf modification, giving rise to its signature bilobed snap-traps. In this mini-review we discuss morphological and biomechanical aspects of *D. muscipula* adaptations to performing the fast-snapping movement. We also give a brief overview of the motion sequences performed by *D. muscipula* during short and long hunting cycles and discuss the potential failure of the trap lobes during reopening.

## Morphological adaptations and the triggering mechanism

The snap-traps of *D. muscipula* consist of two lobes kinematically separated by the midrib, maintaining a concave shape in the “ready to snap” configuration—as seen from the outside of the trap. The snap-traps are able to almost instantaneously change the lobe geometry to convex upon triggering of the snap-through motion (Poppinga and Joyeux, 2011). Each half of the bilobed trap is fitted with 3–5 specialized mechanosensory hairs, arranged in a semi-triangular shape on the inner lining of each lobe (Stuhlman, 1948; Yang et al., 2010). The trigger hairs are highly sensitive, allowing detection of motile prey as minute as ants entering the trap (Jacobson, 1965; Scherzer et al., 2019; Saikia et al., 2020, 2021). *Dionaea muscipula* relies on hapto-electric signaling to trigger trap closure, where the mechanical stimulation of the trigger hair generates a receptor potential (RP) which in turn can elicit an action potential (AP) and associated calcium flux (Jacobson, 1965; Escalante-Pérez et al., 2011; Volkov, 2019; Scherzer et al., 2022). In general, the sensory hairs require two mechanical stimuli within a time window of ~30 s in order to reach a threshold AP and consequently evoke trap closure (Macfarlane, 1892; Brown and Sharp, 1910; Juniper et al., 1989; Burri et al., 2020). However, it is also possible to trigger the traps to snap with a single, prolonged stimulus generating two APs (Burri et al., 2020), or without touching the mechanosensitive hairs at all, by raising temperature of the plant above 40°C, thus inducing temperature-dependent autonomous AP firing (Fabricant et al., 2021), by using bioactive metabolites (Ueda et al., 2010) or chemicals such as H_2_O_2_, HNO_3_ (Volkov, 2019) or NaCl (Böhm et al., 2016b), plasma-generated reactive oxygen and nitrogen species (Volkov et al., 2019) or by applying direct electrical stimulation to the midrib (Volkov et al., 2007, 2008). The 30 s window for mechanical stimulation is linked to the ability of the plant to “memorize” stimuli (Juniper et al., 1989; Volkov et al., 2008, 2009)—an ability closely tied with calcium signaling (Suda et al., 2020). A second AP generated outside of the 30 s window would not be sufficient to reach the threshold calcium ion concentration anymore, as the signal from the first AP would have already degraded by then (Hodick and Sievers, 1988; Hedrich and Neher, 2018).

## Hunting cycle

**Figure 1:**
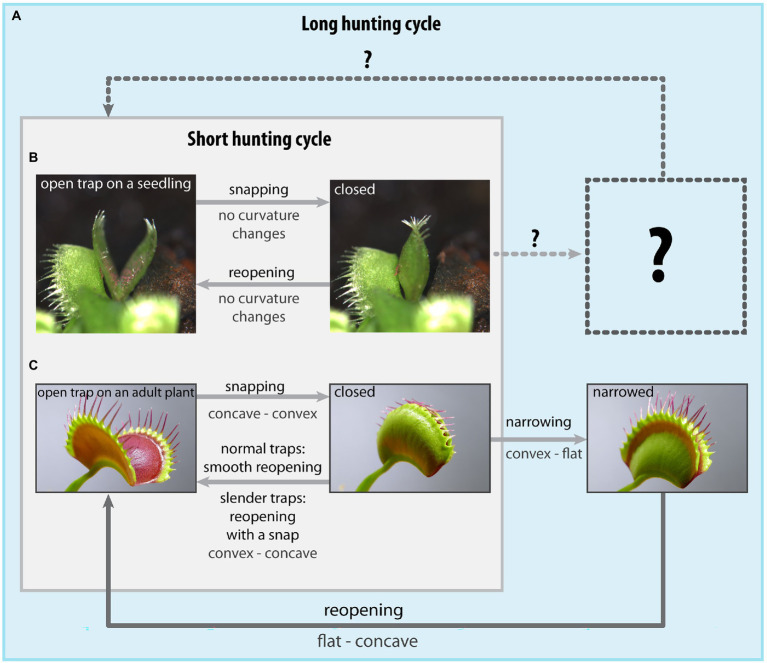
Diagram depicting the range of motions and geometry changes performed by *Dionaea muscipula* snap-traps during short and long hunting cycles. **(A)** – The long hunting cycle in juvenile and adult plants includes successful capture, retention and digestion of prey, whereas during the short hunting cycle, **(B,C)** no prey is successfully captured, leading to an immediate trap reopening after snapping. The mode of trap reopening in traps from adult plants depends on trap geometry, with slender traps sometimes incorporating a snap-through transition. **(B)** – A trap from a juvenile plant in the “ready-to-snap” and closed configurations, **(C)** – A trap from an adult plant in the “ready-to-snap” and closed configurations. Snapping in traps of juvenile plants is not characterized by the snap-buckling instability, otherwise speed-boosting the motion of the adult traps hinting at different closing mechanics. The subsequent trap actuation and deformation processes involved in prey retention and digeston in traps of the juvenile plants are completely unknown so far (indicated by question marks). Images of traps from the juvenile plants were adapted from Poppinga et al. (2016), images of traps of the adult plants were adapted from Bauer et al. (2021).

Following the initial triggering and snap closure, *D. muscipula* can either reopen after failing to catch prey (short hunting cycle, Figures 1B,C) or, if a prey item is successfully captured, initiate formation of a digestive cavity in which the prey can be absorbed (long hunting cycle, Figure 1A; Volkov et al., 2011). In both cases, the plant executes a complex motion sequence in preparation for a new hunting cycle, sharing the initial steps involved in trap triggering and the fast snapping motion as detailed below.

## Biomechanics of snap-trap closure in traps of adult plants

Traps of the adult *D. muscipula* plants can conform to two different snapping scenarios: with both lobes moving synchronously or asynchronously—with one lobe snapping before the other (Poppinga et al., 2016). *Dionaea muscipula* is also capable of performing the fast trap closure underwater (Poppinga et al., 2016), enabling aquatic prey capture when the plants become submerged (Roberts and Oosting, 1958; Schnell, 2002; Bailey and McPherson, 2012).

Once triggered, the doubly curved *D. muscipula* snap-traps undergo hydraulically driven lobe deformation (Hedrich and Neher, 2018; Scherzer et al., 2019; Bauer et al., 2021) generating a buckling instability (Forterre et al., 2005; Poppinga and Joyeux, 2011; Sachse et al., 2020). Thus accumulated elastic energy is then released during snap-through of the trap, where the geometry of the trap lobes changes from concave to convex (Figures 1B,C) due to snap-buckling—a motion performed between 100 and 300 ms (Forterre, 2013). The impact of the trap lobes during snapping generates a closing force of 0.149 N and pressure of 41 kPa between the adjacent lobe rims (Volkov et al., 2013). The snapping speed does not appear to be correlated with trap length (Poppinga et al., 2016) as initially suggested by Forterre et al. (2005). The snapping process is governed by a complex biochemical cascade triggered by the APs spreading through the trap lobe due to the mechanical stimulation of the trigger hairs (Hedrich and Neher, 2018; Volkov, 2019). The ecophysiological, biochemical, molecular and evolutionary aspects of the prey trapping process are thoroughly reviewed in, e.g., Fleischmann et al. (2018); Adamec et al. (2021); Böhm and Scherzer (2021); Hedrich and Fukushima (2021); and Freund et al. (2022). Although the fast snapping cannot be explained by hydraulic actuation alone (Skotheim and Mahadevan, 2005; Colombani and Forterre, 2011) water displacement causing differential changes in the trap tissues was identified as a factor playing a critical role in lobe deformation during snap closure, therefore making the full trap turgescence a prerequisite for a normal execution of the snap-through motion. Dehydrated traps are characterized by an increased angle between the trap lobes (by *ca.* 10%) and an inability to snap, indicating that turgescence is vital in maintaining the “ready to snap” conformation, as it was possible to recover the motility of the traps by allowing the plant to rehydrate (Sachse et al., 2020). Interestingly, the pattern and speed of the snapping process can also be affected by the position of the trigger hair responsible for generation of the RPs and APs (Stuhlman, 1948), and consequently the Ca^2+^ wave involved in plant signaling (Suda et al., 2020). Previous theoretical models of the snap-trap closure operating within a simplified, two-layered system provided crucial theoretical assumptions, such as introduction of the concept of the elastic energy, which upon triggering of the trap and causing leaf relaxation enters a new equilibrium state (Forterre et al., 2005), and prestress arising from mechanically connected hydraulic cell layers characterized by different hydrostatic pressure (Markin et al., 2008). In the latter model, the authors propose a hydroelastic curvature mechanism, where weakly curved, thin and elastic shells composed of an inner and an outer hydraulic layer behave in a manner similar to a bilayer, where if one layer undergoes in-plane shrinking, the other one responds by expanding and *vice-versa*, thus changing the conformation of the whole leaf. The two hydraulic layers in this model are assumed to be connected *via* pores, which open following trap triggering, causing a rapid surge of water between the layers and relaxation of the leaf as it reaches the new equilibrium conformation. Building further on these models, Sachse et al. (2020) were able to show, that the “ready-to-snap” configuration is linked with the hydrostatic pressure in the trap tissues generating a threshold value of prestress in the leaf (the loading), which is then rapidly released as elastic energy during the snap-buckling of the closing *D. muscipula* trap. Contrary to previous theories suggesting that the middle mesophyll layer does not play an active role in the actuation of the snapping motion (Hodick and Sievers, 1989; Fagerberg and Allain, 1991), Sachse et al. (2020) concluded that the mesophyll can act as a lever between the inner and outer epidermises, as corroborated by their *in situ* experiments as well as Finite Element (FE) simulations. Based on their FE simulations combined with Digital Image Correlation (DIC) data allowing direct observation of the evolution of strain during the snapping motion *in situ*, Sachse et al. (2020) propose, that the snap-trap closure relies on the outer epidermis expanding perpendicular to the midrib in the central region of the trap, while the inner epidermis undergoes shrinking at the same time.

## The mechanism of trap closure and reopening in seedlings

Even small, few millimeter long traps of *D. muscipula* seedlings are quite capable of prey capture, yet not much is known about the mechanics involved in the relatively slow trap closure and subsequent reopening (Poppinga et al., 2016). The traps of juvenile plants follow a synchronous closure of the trap lobes and—unlike the traps in adult plants—do not undergo lobe curvature inversion during this process (Figure 1B). The angle between trap lobes in traps produced by the seedlings is much smaller than in the traps of adult plants, recorded as 48° and 82° respectively. The fastest snapping duration for a seedling was measured at 4.96 s, a time significantly slower than that of a trap on an adult plant, which only takes 100–300 ms to snap shut. Therefore, it was proposed that the trap closure in seedlings is actuated strictly hydraulically, acquiring the capability to perform snap-buckling later on during the plant development (Poppinga et al., 2016).

## Biomechanics of trap reopening in snap-traps of adult plants

Trap reopening after fast snap-closure without prey capture is the final stage of the short hunting cycle (Figure 1C). Traps usually reopen within 16–44 h, with smaller morphotypes reported to have shorter median reopening times than the larger ones (Volkov et al., 2011; Poppinga et al., 2016; Durak et al., 2022). Each trap can only reopen a finite amount of times (usually 3–12), before losing functionality (Bailey and McPherson, 2012). For a long time, the exact mechanism of trap reopening was rather elusive and assumed to be a slow, relatively homogenous process reliant on an interplay between cell turgor and localized cellular growth and/or expansion (Fagerberg and Howe, 1996; Yang et al., 2010; Volkov et al., 2011). However, previous attempts to assess changes in cell length during trap reopening cycle yielded inconclusive results (Fagerberg and Howe, 1996). Trap reopening can follow several scenarios, with both trap lobes bending outwards homogenously (Volkov et al., 2011; Poppinga et al., 2016) or asynchronously, with one lobe opening before the other, or with a distinct “rim-pop” at the beginning of the reopening sequence (Durak et al., 2022). The “rim-pop” was reported to occur right at the edge of both normal-sized and large traps alike during the initial reopening stage, and is most likely caused by the sticky nectar deposited around the rim of the trap (Mozingo et al., 1970; Juniper et al., 1989), or can result from the resistance arising from the marginal cilia (“teeth”) sliding apart. Whether the lobes open synchronously or not, the reopening process on the global scale can either commence in a smooth, homogenous fashion or with a snap-buckling step towards the end of the reopening sequence (Durak et al., 2022). Upon closer investigation of the reopening scenarios, Durak et al. (2022) concluded that the observed behavior is highly dependent on the trap size and slenderness. In their study, they show that traps below 3 cm in length [normal-sized (N) traps] open exclusively *via* a smooth, homogenous reopening sequence in a synchronous outwards lobe bending motion. In contrast, traps ≈ 3–4.3 cm [large (L) traps] can reopen not only in a synchronous or asynchronous manner, but 3.85% of the L traps were additionally able to incorporate a reverse snap-through step into the reopening sequence. Since the snap-through process was previously considered energetically costly and even impossible to perform due to the mechanics involved (Fagerberg and Howe, 1996; Poppinga et al., 2016), Durak et al. (2022) set out to investigate the potential of the trap morphometrics playing a role in determining whether a trap is capable of going through the snap-through during reopening or not. As comparison of the distribution of the evolving strain patterns between N and L traps did not yield any significant differences, Durak et al. (2022) looked into the overall slenderness of the traps within these two morphotype categories. They found, that L traps were characterized by higher overall slenderness and concluded that due to the high spread of the slenderness values within a given morphotype, it is possible that only the most slender traps (3.85% of the L trap tested) are capable of reverse snap-buckling. The study further corroborated that, even though there is a significant overlap in the strain patterns affecting inner and outer epidermis during fast closure and trap reopening, the trap reopening is by no means a simple emulation of the fast closure in reverse. The authors further stated that although trap slenderness can to some extent explain the differences in trap reopening behavior between the N and L traps, it is likely that there are other, physiological or mechanical factors involved in obstruction of the snap-through during the reopening cycle, which should be investigated in the future studies on this topic.

## Trap reopening in seedlings

The information on trap reopening in *D. muscipula* seedlings is very limited with only one study by Poppinga et al. (2016) investigating it in more detail, where it is shown that the seedling traps reopened from a closed, “no prey” configuration *via* a slow, homogenous process without any sudden changes to the lobe movement. The authors speculated that seedlings could potentially reopen by reversing the hydraulic process likely underlying the slow snapping in seedlings—a mechanism potentially allowing to avoid growth-related obstruction of the snapping process affecting adult plants.

## Trap sealing and formation of the digestive cavity: The long hunting cycle

Once a prey item is successfully captured by the snap-trap, stimulation of the mechanoreceptors by the struggling prey activates the jasmonate (JA) signaling pathway as well as expression of hydrolases necessary for prey digestion (Escalante-Pérez et al., 2011; Böhm et al., 2016a). JA are hormones involved in plant stress response (Wasternack and Hause, 2013), and are also involved in formation of the secretory vesicles in carnivorous plants such as *D. muscipula* (Scherzer et al., 2017). The formation of the “green stomach” involves locking of the marginal cilia, subsequent lobe flattening aka. narrowing and hermetic sealing of the trap (Figure 1A; Volkov et al., 2011), with the process requiring continuous mechanical and chemical stimulation by the prey in order to trigger the signaling pathways essential for digestion (Libiaková et al., 2014; Böhm et al., 2016a; Scherzer et al., 2017). The escaping force necessary for the prey to be able to get out of the trap increases from 0.188 N following snap-closure all the way up to 4 N as the trap narrows around the prey (Volkov et al., 2013). The constriction force generated on the prey as the trap narrows reaches 0.45 N, with the maximum constriction pressure peaking at 9 kPa (Volkov et al., 2013). Following the digestion process, the trap reopens once again changing the lobe conformation from convex to concave, taking an average of 7 days for the cycle to complete (Volkov et al., 2011). Although the biochemical pathways underlying green stomach formation are relatively well studied in adult plants, very little is known about the mechanics involved in the elaborate shape shifting of the traps in adult and juvenile plants during prey digestion, as well as reopening after the process is complete.

## Costs of a failed hunting cycle

Failing to capture prey during a hunting cycle is bound to incur costs on the plant itself. These costs include energy expenditure on the complex, biochemically mediated snapping motion (Jaffe, 1973; Maurer et al., 2020) as well as the resulting transient decrease in ΦPSII after the mechanical triggering (Pavlovič et al., 2011), combined with the relatively high costs of oxygen-based dark respiration in relation to the photosynthetic rate (Adamec, 2010; Pavlovič et al., 2011). Once the trap snaps, it takes on average 16–44 h before it reopens (Volkov et al., 2011; Poppinga et al., 2016; Durak et al., 2022) during which time the trap is unable to contribute towards active prey capture. In addition to that, the reopening process itself is bound to generate further costs to the plant, even if the process relies on transpiration as the least metabolically costly mode of water transport (Fricke, 2017). However, in our view, the highest costs of a failed hunting cycle are: (1) the imminent loss of trap functionality following several snapping/reopening cycles and (2) trap breakage during an attempt to reopen the snap-trap. It was observed, that about 3.44% of L traps undergo spontaneous breakage during trap reopening (Durak et al., 2022). The tears form in the trap area near the midrib, in a semi-parallel, diffuse or crescent pattern, affecting one or both lobes and penetrating the tissue deep into the mesophyll. Traps affected by these deep tears lose the ability to reopen and thus are no longer capable of trapping prey. Trap breakage was reported to affect larger (>2.5 cm in length), more slender traps with a lower length-to-height ratio, indicating the existence of a quasi-size limitation on the continuous trap functionality (Durak et al., 2022). Carnivorous plants are classed as “slow and tough” on the universal leaf economics spectrum (Wright et al., 2005; Shipley et al., 2006; Osnas et al., 2013) and are characterized by relatively low trap construction costs and very low maximum leaf photosynthetic rates (Karagatzides and Ellison, 2009). The payback time, defined as the amount of time necessary to compensate the costs of growing a new trap in terms of net carbon generated *via* photosynthesis (Poorter et al., 2006) is relatively high, exceeding 1,000 h in *D. muscipula* (Karagatzides and Ellison, 2009). However, due to its specific habitat, *D. muscipula* does not experience light or water limitation, and therefore these costs can be offset by the enhancement of photosynthesis due to influx of the limiting nutrients—N and P—absorbed from prey (Givnish et al., 1984; Kruse et al., 2014). Since the plant tends towards redistribution of nutrients and trap growth/renewal following prey capture (Kruse et al., 2014), it is reasonable to assume that spontaneous trap breakage can incur a significant cost to the plant when replacing the affected leaf in order to maintain its ability to effectively hunt for prey necessary for optimum nutrition. It is also noteworthy, that trap injury due to breakage induces at least partial formation of a digestive cavity, as the plant is unable to discern between injury and prey capture (Pavlovič et al., 2017). Additional costs stemming from this phenomenon require further assessment, as the digestive process is known to be metabolically costly, and in this case, cannot be offset by successful prey capture.

## Conclusion and perspective

Although snap-closure and trap reopening after fast snapping are relatively well investigated from both theoretical and experimental point of view, the mechanics of the “green stomach” formation as well as reopening of the trap following prey digestion are still poorly understood. Similarly, biomechanics involved in the slow closure and reopening of the geometrically distinct traps in seedlings as well as the effect of the trap size on trap closure should be addressed in more detail. We would also like to highlight the importance of providing detailed specifications of the size class and characteristics of the plant strain used in the future studies, as this information is not provided in many research papers available to date, thus rendering the results very hard to compare and interpret. Based on the current models as well as empirical data, it is clear that size and particular geometrical characteristics of the plants investigated play a crucial role in trap behavior both during the snap-closure as well as the reopening process. The concept of the potential size limitation of snap-traps could provide interesting new information not only on the biomechanical principles dictating the growth of the plant, but could also provide insight into evolution of plants capable of fast thigmonastic motion in terms of size distribution of the motile parts of the plants with a carnivorous habit, which are not too prone to disastrous trap failure during reopening. As to the latter aspect, it would be interesting to investigate the existence of a tradeoff between the advantage of a potentially faster trap reopening involving snap-buckling mechanism and the (inevitable) possibility of trap failure during this process.

## Author contributions

GD analyzed, interpreted, and reviewed the research articles, prepared the figures, and drafted the article. TS and SP designed the research framework, acquired the funding and critically revised the manuscript. All authors contributed to the article and approved the submitted version.

## Funding

GD and TS acknowledge the Ministry of Science, Research and the Arts Baden-Wuerttemberg for financial support of the project “Bio-inspirierte elastische Materialsysteme und Verbundkomponenten für nachhaltiges Bauen im 21ten Jahrhundert (BioElast).” SP and TS further acknowledge funding by the Deutsche Forschungsgemeinschaft (DFG, German Research Foundation) under Germany’s Excellence Strategy – EXC-2193/1 – 390951807.

## Conflict of interest

The authors declare that the research was conducted in the absence of any commercial or financial relationships that could be construed as a potential conflict of interest.

## Publisher’s note

All claims expressed in this article are solely those of the authors and do not necessarily represent those of their affiliated organizations, or those of the publisher, the editors and the reviewers. Any product that may be evaluated in this article, or claim that may be made by its manufacturer, is not guaranteed or endorsed by the publisher.
